# Imposter Syndrome in Dentistry: Prevalence, Contributing Factors, and Educational Implications: A Narrative Review

**DOI:** 10.7759/cureus.100384

**Published:** 2025-12-29

**Authors:** Abdulmohsen A Mohammed, Ahmad Al-Omar

**Affiliations:** 1 College of Dentistry, King Saud University, Riyadh, SAU; 2 Surgery, King Saud University, Riyadh, SAU

**Keywords:** dental education, dental students, imposter syndrome, perfectionism, professional identity

## Abstract

Imposter syndrome is a psychological phenomenon characterized by persistent self-doubt and fear of being exposed as incompetent despite objective evidence of ability and achievement. Although it has been widely studied across health professions education, its presence and implications within dental education have received comparatively less focused attention. The demanding nature of dental training, marked by early clinical exposure, technical precision, and continuous evaluation, may place dental learners at increased risk of developing imposter feelings. This narrative review synthesizes existing literature published between 2000 and 2025 examining imposter syndrome in dentistry and related health professions education, with a focus on prevalence, psychological determinants, educational and environmental contributors, and associated consequences. The reviewed literature indicates that imposter syndrome is common among dental students and healthcare trainees and is strongly associated with perfectionism, fear of failure, low self-esteem, and heightened academic and clinical stress. Educational factors such as competitive learning environments, inconsistent feedback, and high-stakes clinical assessments further exacerbate imposter experiences. These factors have been linked to reduced self-confidence, avoidance of complex clinical tasks, psychological distress, and challenges in professional identity formation. Imposter syndrome represents a significant but often underrecognized challenge in dental education, with implications extending beyond emotional discomfort to influence learning behaviors, clinical engagement, and overall well-being. Addressing this phenomenon requires increased institutional awareness, supportive educational environments, and structured mentorship strategies, while future research should prioritize longitudinal outcomes and the development of targeted interventions to support dental learners throughout their training.

## Introduction and background

Imposter syndrome, first identified by Clance and Imes in 1978, refers to a psychological phenomenon characterized by a persistent belief of personal inadequacy and fear of being exposed as incompetent, despite objective evidence of ability and achievement [1]. Over the past four decades, research has demonstrated that imposterism is not confined to any single demographic; rather, it affects learners and professionals across diverse academic and clinical settings, contributing to self-doubt, fear of failure, perfectionistic tendencies, and emotional distress [2-4]. Within health professions education, this phenomenon appears particularly pronounced. Systematic reviews and meta-analyses indicate that healthcare providers and students report some of the highest global prevalence rates of imposter symptoms, which are consistently associated with psychological distress, anxiety, reduced self-efficacy, and decreased intention to persist in clinical training [5-8]. These experiences often intensify during key transitional stages, such as entry into clinical rotations or the onset of residency training, when performance becomes more visible and professional identity is still forming [9-11].

Within dentistry, imposter syndrome carries distinct educational and psychological implications. Dental students are exposed early to patient care, undergo continuous evaluation of manual dexterity, and face high expectations for technical precision, conditions that may heighten social comparison, fear of appearing incompetent, and reluctance to seek feedback [6,12]. Studies from the Middle East, Europe, and Asia report that imposter feelings among dental learners are associated with increased anxiety, reduced confidence in clinical procedures, and avoidance of challenging tasks [13-15]. Importantly, these experiences are shaped not only by individual characteristics such as perfectionism and achievement-related stress [7], but also by educational environments characterized by competitive academic climates, limited mentorship, and feedback cultures that may unintentionally reinforce self-doubt [8,16]. Despite growing scholarly interest, research on imposter syndrome within dentistry remains fragmented compared to medicine and nursing, with relatively few integrative analyses examining its causes, manifestations, and consequences across the dental training continuum. Additionally, internal attitudes toward specific patient groups, such as elderly patients, have been shown to influence dental students’ satisfaction, perceived clinical competence, and self-confidence during care delivery [17].

Given these gaps, a focused narrative review is warranted to synthesize current evidence, contextualize imposter syndrome within dental education, and clarify how psychological, educational, and institutional factors interact to shape learners’ experiences. Accordingly, this review aims to (1) consolidate contemporary literature on imposter syndrome in health professions education with particular emphasis on dental students and dental faculty; (2) identify key themes related to prevalence, psychological determinants, and educational consequences; and (3) highlight areas requiring further investigation to inform the development of targeted, dentistry-specific interventions.

## Review

Materials and methods

This study was conducted as a narrative (non-systematic) literature review aiming to synthesize existing evidence related to imposter syndrome in dentistry and health professions education. Relevant literature was identified through searches of electronic databases, including PubMed, Scopus, Google Scholar, and ProQuest, covering publications from 2000 to 2025. Search terms included combinations of “imposter syndrome,” “imposter phenomenon,” “dental education,” “dental students,” and related psychological and educational concepts.

An iterative and purposive screening approach was employed, in which titles and abstracts were reviewed to identify literature relevant to imposter-related experiences within healthcare and dental education contexts. Full-text articles were consulted when necessary to support conceptual understanding. Studies were selected based on their relevance to the topic, contribution to educational or psychological insight, and clarity of conceptual or methodological focus. Only English-language publications were included.

Based on these criteria, a total of 30 studies were included, encompassing cross-sectional survey studies, empirical observational studies, systematic reviews, meta-analyses, and narrative reviews addressing imposter syndrome, psychological stress, perfectionism, and educational outcomes among dental and other healthcare learners. The majority of included studies focused on undergraduate or postgraduate healthcare learners, with a subset specifically examining dental students and dental faculty, allowing comparison of consistent and conflicting findings across different educational and professional contexts.

Given the narrative nature of this review, no formal risk-of-bias assessment or statistical synthesis was performed. Instead, included studies were examined thematically to identify recurring patterns, areas of convergence or divergence, and gaps in the existing literature. These themes informed the structure of the review and guided the interpretation of the findings. Foundational and seminal articles published prior to 2000 were used selectively to provide historical and conceptual context and were not included in the formal study sample analyzed in the Results section.

Results

Prevalence of Imposter Syndrome in Dental and Health Professions Education

The findings of this review demonstrate that imposter syndrome is a pervasive and multifactorial phenomenon across health professions education. Global meta-analytic evidence confirms the high prevalence of imposterism among healthcare providers [2,18], consistent with earlier systematic reviews documenting its widespread presence in medical and academic populations. Studies involving medical, dental, and allied health students consistently report strong associations between imposter feelings and psychological distress, anxiety, and reduced psychological well-being [5,9,10]. Within dentistry, imposterism appears particularly prevalent due to early clinical exposure, precision-based procedural requirements, and continuous performance evaluation inherent to dental training [12,19,20-24].

Psychological Determinants

Psychological determinants play a central role in the development of imposter syndrome. Perfectionism, identified as one of the strongest predictors of imposter tendencies, has been repeatedly documented among medical, dental, nursing, and pharmacy students [7]. Individuals with pronounced perfectionistic traits tend to interpret minor mistakes as evidence of incompetence, thereby reinforcing persistent self-doubt. In addition, studies conducted in Saudi Arabia, Oman, and South Asia demonstrate significant associations between imposterism, low self-esteem, fear of judgment, and increased intention to withdraw from health professions programs [14-18].

Educational and Environmental Factors

Educational and environmental factors further intensify imposter experiences. Competitive academic climates, unclear expectations, inconsistent feedback, and limited access to mentorship have all been identified as contributing triggers [8,12,13,16]. Studies conducted in Saudi Arabia, Oman, and South Asia suggest that these pressures may be amplified within rapidly evolving educational systems, where heightened academic demands and competitive training environments influence learner confidence and professional identity formation [14-18]. The growing body of orthodontic and dental research within Arab League nations further reflects these evolving academic pressures and competitive educational climates, which may contribute to the psychological experiences reported among dental learners [25]. Evidence from simulation-based training, such as studies conducted in pediatric residency programs, suggests that structured and supportive practice environments may help reduce imposter feelings by enhancing clinical preparedness [15]. However, within dental education, mentorship quality remains variable, and feedback practices often emphasize error detection rather than developmental support, potentially reinforcing imposter experiences [21-23].

Academic and Professional Consequences

The reviewed literature also highlights substantial functional and professional consequences associated with imposter syndrome. Reported outcomes include avoidance of complex clinical tasks, excessive overpreparation, reluctance to seek guidance, reduced self-efficacy, and heightened performance anxiety [5,10-12]. Over time, these behaviors may contribute to impaired professional identity formation, decreased readiness for independent clinical practice, and a reduced likelihood of assuming leadership roles within healthcare environments [11,16].

Intervention Strategies and Research Gaps

Despite increasing recognition of the problem, intervention-focused research remains limited. While mentorship-based initiatives, reflective discussion groups, and psychoeducational strategies have shown preliminary promise in mitigating imposter feelings [22,23], dentistry lacks structured and validated interventions tailored specifically to its educational context. Recent scoping and narrative reviews emphasize the need for longitudinal and discipline-specific research to evaluate the effectiveness of educational reforms and support strategies in addressing imposter syndrome [23]. Additional dentistry-focused studies further support the high prevalence of imposter syndrome among dental students [19,20-24] and dental faculty [26-30], highlighting associations with academic stress, clinical confidence, and professional self-perception across different stages of dental training.

Discussion

The findings of this narrative review suggest that imposter syndrome in dental education emerges from a complex interaction between individual psychological characteristics and the structural demands of dental training. The nature of dental education, marked by early patient exposure, high levels of personal clinical responsibility, and continuous performance evaluation, appears to create conditions in which imposter feelings may be amplified. These structural factors interact with internal traits such as perfectionism, fear of failure, and low self-confidence, reinforcing persistent self-doubt despite demonstrated competence [1,7,8,12].

Educational environments also play a critical role in shaping imposter experiences. Variability in mentorship quality, feedback styles that emphasize error identification over developmental growth, and highly competitive academic cultures may unintentionally exacerbate imposter tendencies. In dentistry, where procedural precision and patient outcomes are closely scrutinized, learners may be particularly vulnerable to interpreting normal learning challenges as personal inadequacy. These findings align with broader literature across health professions education while highlighting discipline-specific stressors unique to dental training [8,10,12,13,16].

The implications of imposter syndrome extend beyond psychological discomfort. Persistent imposter feelings may influence learning behaviors, clinical engagement, and professional identity formation. Dental trainees experiencing imposterism may demonstrate reluctance to seek feedback, avoidance of complex procedures, and excessive self-criticism, potentially affecting skill acquisition and confidence development. Over time, these patterns may hinder readiness for independent clinical practice and limit engagement in leadership or academic roles [5,10-12,16,18].

Conceptual Understanding of Imposter Syndrome in Dental Education

Imposter syndrome can be conceptualized as a context-dependent phenomenon shaped by both learner factors and the educational culture in which competence is evaluated. Foundational descriptions emphasize that imposter feelings may persist even among high-achieving individuals despite objective performance, particularly when success is attributed to external factors rather than ability [1,3]. Within health professions education, evidence repeatedly links imposterism to perfectionism and achievement-related cognitions, which can intensify self-monitoring and negative self-appraisal in high-stakes learning environments [7,9]. This framing supports interpreting imposter experiences in dentistry not as isolated individual pathology, but as an interaction between psychological predispositions and training structures that emphasize precision, evaluation, and continuous performance comparison [7-9,12].

Educational and Institutional Interventions

Although dentistry-specific intervention studies remain limited, the available evidence across healthcare education suggests several institutional approaches that may reduce imposter experiences. Supportive mentorship models, balanced feedback practices, and programs that normalize challenge and error during procedural learning may help protect learners from interpreting normal developmental struggles as personal inadequacy [22,23]. Structured simulation-based learning may also improve preparedness and confidence, which could indirectly reduce imposter feelings during early clinical exposure [15]. In dentistry, aligning feedback culture toward developmental coaching, rather than exclusively error detection, may be particularly important given the procedural and performance-sensitive nature of clinical training [23,24]. Practical guidance and professional commentaries within dentistry additionally emphasize the value of open discussion, supportive supervision, and proactive learner support in mitigating imposter experiences [26,29]. While many intervention models originate from broader health professions education, their core mechanisms, such as mentorship continuity, formative feedback, and structured skills development, are highly transferable to dental education due to shared characteristics, including early clinical responsibility, performance-based assessment, and close faculty supervision. Adapting these approaches within dental curricula may therefore offer a practical and contextually relevant strategy to mitigate imposter experiences among dental learners.

Future Directions and Research Implications

The current literature in dentistry remains dominated by descriptive and cross-sectional designs, limiting causal inference and the ability to evaluate the effectiveness of proposed educational reforms [10,18]. Future research should prioritize longitudinal designs to track imposter feelings across the transition from preclinical to clinical training and into independent practice and should incorporate validated measurement tools appropriate for large-scale and repeated assessment [14]. Intervention-focused studies tailored to dentistry are also needed, particularly those evaluating structured mentorship frameworks, feedback reform, and wellness-oriented curricula using rigorous outcomes and follow-up [23]. Expanding research to include dental faculty is equally important, as faculty imposterism may reflect wider institutional and cultural dynamics that shape learner experiences and professional identity formation [30].

Evidence from dentistry-focused literature emphasizes that imposter experiences are not limited to students but also extend to dental educators and faculty members, suggesting a broader cultural and institutional influence within dental education environments [19,26,27,30].

Limitations

This review has several limitations. As a narrative review, the selection and interpretation of studies may be subject to selection bias, and the findings are dependent on the quality and scope of the included literature. Additionally, much of the existing evidence relies on self-reported measures of imposter syndrome and cross-sectional study designs, limiting causal inference. Finally, dentistry-specific intervention studies remain scarce, highlighting the need for further research in this area.

## Conclusions

Imposter syndrome represents a prevalent and multifaceted challenge within dental education, shaped by the interaction between individual psychological traits and the demanding structure of dental training. The evidence synthesized in this review highlights how factors such as perfectionism, evaluative learning environments, and early clinical responsibility contribute to persistent self-doubt among dental learners.

Rather than reflecting individual inadequacy, imposter syndrome appears to be strongly influenced by educational context and institutional culture. Recognizing imposterism as a systemic issue rather than a personal failing is therefore essential for fostering supportive learning environments within dental education. Addressing this phenomenon may improve learner well-being, enhance clinical engagement, and support healthy professional identity development. Future efforts should prioritize the development and evaluation of dentistry-specific interventions, including structured mentorship models, constructive feedback frameworks, and educational strategies that normalize challenge and error during skill acquisition, in order to promote sustainable professional growth across different stages of dental training.
